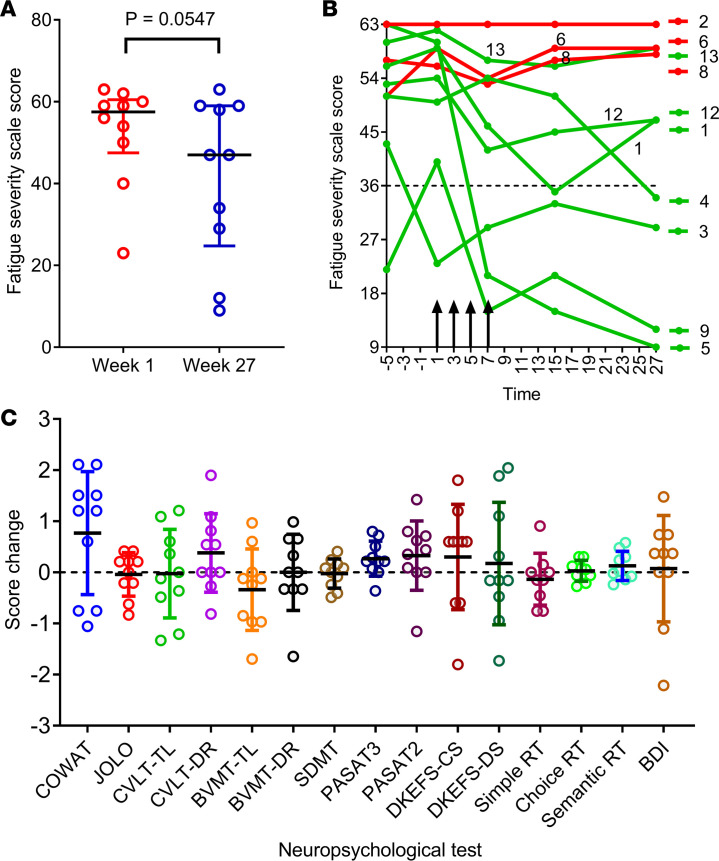# Epstein-Barr virus–specific T cell therapy for progressive multiple sclerosis

**DOI:** 10.1172/jci.insight.144624

**Published:** 2020-10-15

**Authors:** Michael P. Pender, Peter A. Csurhes, Corey Smith, Nanette L. Douglas, Michelle A. Neller, Katherine K. Matthews, Leone Beagley, Sweera Rehan, Pauline Crooks, Tracey J. Hopkins, Stefan Blum, Kerryn A. Green, Zara A. Ioannides, Andrew Swayne, Blake T. Aftab, Kaye D. Hooper, Scott R. Burrows, Kate M. Thompson, Alan Coulthard, Rajiv Khanna

Original citation: *JCI Insight*. 2018;3(22):e124714. https://doi.org/10.1172/jci.insight.124714

Citation for this corrigendum: *JCI Insight*. 2020;5(20):e144624. https://doi.org/10.1172/jci.insight.144624

During the preparation of this manuscript, errors relating to data for BVMT-TL, BVMT-DR, PASAT3, and PASAT2 were inadvertently introduced into Figure 3C. Several of the data points have changed, but there are only minimal changes in the mean values and standard deviations. In addition, in Results, in the section *Clinical outcomes following T cell therapy*, the *P* value for PASAT3 is incorrect. However, after applying Bonferroni’s correction for multiple comparisons, the new *P* value still does not reach significance. The correct sentence and figure part are below. The online HTML and PDF versions of the manuscript have been updated to reflect these changes.

Statistically significant, or nearly significant, increases in attainment were observed at week 27 across two measures, the Controlled Oral Word Association Test (COWAT, a verbal fluency test) and the Paced Auditory Serial Addition Test (PASAT3, a working memory/processing speed task) (*P* = 0.074 and *P* = 0.0356 respectively, paired 2-tailed *t* test); however, after applying the Bonferroni correction for multiple comparisons, these *P* values no longer reached significance.

The authors regret the errors.

## Figures and Tables

**Figure 3:**